# Observations on the Supposed Frequency of Ulceration of the Os and Cervix Uteri

**Published:** 1850-11

**Authors:** 


*Observations on the Supposed Frequency of Ulceration of the Os and Cervix Uteri.* By Tyler Smith, M. D., Lecturer on Midwifery at the Hunterian School of Medicine; one of the Physicians to Queen Adelaide's Lying-in Hospital.

Mr. Whitehead, of Manchester, in his work on "Abortion and Sterility," states, that of 2000 women whose cases he investigated on their application to the Manchester Lying-in Hospital, "1116 had the whites at the time the inquiry was made, and a considerable number more had suffered under a similar ailment at some former period. In 936, or eighty-three per cent., the discharge bore undoubted evidence of the presence of pus, or of sanies; and in some instances it was more or less mixed with blood." Mr. Whitehead traces these discharges to "disease of the lower part of the uterus, this disease being found to exist in almost every instance;" and he further declares, that "this lesion of structure constitutes the true pathological seat of leucorrhœa, and of all its associated phenomena"

Dr. Henry Bennet states, in his recent work on "Inflammation of the Uterus and its Appendages, and on Ulceration and Induration of the Neck of the Uterus," that of three hundred cases presenting "uterine symptoms," among the patients of the Western Dispensary, he found that "243 were suffering from decided inflammatory disease of the cervix, or its cavity; and that in 222 ulceration was present." Thus, in Mr. Whitehead's cases, in 936 out of 1116 cases of leucorrhœa, the discharge was purulent or ulcerative; and in Dr. Henry Bennet's cases 222 out of 300, or more than two-thirds, were also suffering from uterine ulceration. Dr. Bennet states, that the same proportions are preserved in the cases he has treated in private practice.

It is well known that this is widely at variance with the experience of previous observers in this country. Does this discrepancy arise from the superior modes of investigation adopted by the authors I have quoted? or, does it happen from some misapprehension as to what really constitutes ulceration of the os and cervix uteri? Is there simply some mistake about the nature of ulceration; or is the difference explained by the more general use of the speculum?

Practising as a physician accoucheur, I must get the same class of patients as those treated by Mr. Whitehead and by Dr. Bennet. I am in the habit of using the speculum in cases of obstinate leucorrhœa in married females, and I trust with a desire to observe truly and faithfully, but I do not myself find uterine ulceration, at least not what seems to me to warrant this term, so frequently as Dr. Bennet, Mr. Whitehead, and some other gentlemen who have written upon the subject, in leucorrhœal cases—purulent or muco-purulent. I find inflammation, engorgement, induration, excoriation, patches of aphthæ, epithelial abrasion, and granulation often enough, but very seldom what I could call Ulceration, in non-malignant and non-syphilitic cases.

The clearest description I can find of the so-called ulceration, is in Dr. Bennet's work, (pages 102 and 103,) which I take the liberty of extracting, and certain expressions in which I have italicised: it occurs under the heading—

"*Inflammatory Ulceration.*—Inflammation may exist for years in the cervix and its cavity, without giving rise to any other anatomical changes than those which have been enumerated. This, however, is seldom the case. The mucous membrane lining these regions, and more especially that portion of it which is near the os, appears to be *peculiarly liable to take on ulcerative action.* Consequently, the existence of inflammation *in the great majority of instances* is soon followed by the manifestation of the ulcerative process. Ulceration generally appears first round the os, and just within the cavity of the cervix. Many different forms or species of ulceration are described by Continental writers, but, in my opinion, without necessity or advantage. An ulceration occupying the cervix uteri may present all the various modifications which suppurating surfaces offer in any other part of the body, from the minute granulations of a slight abrasion, to the livid vegetations of an unhealthy sore; but these modifications of the ulceration require in reality no division or classification."

"When an abrasion or excoriation only is present, the cervix is generally of a vivid red, and the granulations are often so minute, that it is at first difficult to ascertain whether the mucous membrane is abraded or merely congested, or to perceive the limit of the ulceration when once it has been ascertained to exist. The doubt, however, may be solved by lightly touching the suspected surface with the nitrate of silver. The abrasion immediately assumes a much whiter hue than the region which is merely congested, and its margin becomes well defined and evident. *An abraded or excoriated condition of the mucous surface is generally the form under which ulceration presents itself in the cavity of the cervix,* granulations of any size being seldom met with in this region.

"In its more decided form, ulceration of the cervix uteri is susceptible of presenting every possible variety. The granulations may be firm, of a vivid red hue, scarcely bleeding on pressure, or they may be large, fungous, livid, and bleeding profusely on the slightest touch. These fungous ulcerations are generally connected with torpor of the local circulation: when they are present, the congestion of the vagina and cervix is often very great, of a livid venous character, and the non-ulcerated surface of the cervix may present dilated varicose veins."

Now, this portrait, the general fidelity of which I admit, does not appear to me to warrant the profuse use of the term "ulceration," which in itself, and when applied to other parts of the body, has a definite and unmistakable meaning. It cannot truly be said, that mere abrasions or excoriations caused by irritating leucorrhœal discharges are "ulcerations." We do not call the excoriation which occurs from similar causes in other parts of the body "ulceration." We do not call granulations upon mucous membranes "ulcerations," whether they secrete mucus or pus, or whether they bleed or not upon mechanical irritation. If we analyze this important passage closely, we shall find that granulations at the os and cervix uteri is the strongest fact testified to in support of the theory of "ulceration." Dr. Bennet states, that "an ulceration occupying the cervix uteri *may present all the various modifications which suppurating surfaces offer in any other part of the body,* from the minute granulations of a slight abrasion to the livid granulations of an unhealthy sore." But, immediately afterwards, he negatives this by adding, "*An abraded or excoriated condition* of the mucous membrane is generally the *form* under which ulceration presents itself in the cavity of the cervix, granulations of any size being very seldom met with in this region." Thus, it is evident, that even in the absence of granulations, Dr. Bennet calls "an abraded or excoriated condition" a "form" of "ulceration." From his own descriptions, it is evident that Dr. Bennet classes abrasions, excoriations and granulations together, as forms of ulceration, a proceeding which it appears to me is utterly opposed to all sound pathology. In this way we may explain the great frequency with which ulcerations of the os and cervix uteri is declared to be met with.

If we consider excoriation or abrasion as genuine ulceration, probably no woman ever passes through life without suffering from this form of disease. In the virgin uterus, the circulation is frequently modified by the recurrence of menstruation, ovarian irritation, mental emotion, the varying conditions of the bladder and rectum; and in constitutional ailments, the vaginal and uterine secretions, in common with the other secretions of the body, are frequently depraved. Excoriation and abrasion of the mucous membranes are easily accounted for under such circumstances. Menstruation alone, in the turgidity of the uterus and ovaria, before the catamenial flow is established; in the exudation of blood from the surface of the uterus; and in the perforation of the peritonæal membrane for the elimination of the ovule from the ovary, trenches very nearly upon pathology. The slightest divergence from the ordinary function merges into disease.

In married women, and those who have borne children, other prejudicial causes in addition to these are in operation—such are the mechanical irritation of coitus, the risk of lacerations of the os uteri during the passage of the child in parturition, and the state of the uterine orifice which obtains after labor, and the return of the organ to quiescence. After labor, the orifice of the uterus does not contract smoothly, so as to leave the os uteri regular and even, but it becomes puckered and contracted unevenly. In irritable conditions of the mucous membrane of the uterus and vagina, or in a morbid state of the utero-vaginal secretions, these folds or corrugations are very liable to be chapped or excoriated, and I believe this is often mistaken for ulceration. All these, and other causes which I might enumerate, explain the frequency with which the os uteri deviates, in color, volume, and secretion, from the strictly healthy standard. In fact, we may compare the upper part of the vagina to the fauces, which is seldom found perfectly healthy in any subject who may be examined. Some of the indurations and enlargements of the os and cervix uteri appear to resemble enlarged tonsils, and, like them, to increase in size without any amount of active inflammation.

The granulations which are sometimes found surrounding the os uteri—which may secrete mucus or pus abundantly, and which may bleed on being roughly handled—are, I have no doubt, the result of inflammation; but they resemble *the granular state of the conjunctiva*, rather than the granulations of a true ulcer, the granular os uteri offering no edges or signs of solution of continuity, by which we might satisfactorily declare it to be an ulcer. The *granular os uteri* would be a more correct designation, in such cases, than "ulceration" of the os uteri. Some of the so-called ulcerations appear to be nothing more than patches of thickened epithelium, or portions of the os and cervix, from which the epithelium has been melted away by acrid or irritating secretions. We can imitate this condition of the parts by the slight application of the nitrate of silver—sufficient to affect the epithelial covering, but not sufficient to injure the mucous membrane beneath.

It appears to me that we can neither receive the existence of excoriation or abrasion; of granulation or fungous growths; the secretion of pus or muco-purulent matter; as affording undeniable evidence of the existence of "ulceration" of the os and cervix uteri. We must try ulceration in this part of the body by the same tests which we apply to ulcers in other parts of the economy. We must look for a solution of continuity, with a secreting surface, separated from the healthy structures, having defined edges, everted or inverted,—for an ulcer, in fact, in the common pathological meaning of the term. We find ulcers having these characters in the air-passages, mouth, stomach, intestines, bladder, and other mucous surfaces. There is no mistaking the characters of an intestinal ulcer after dysentery, and there ought to be no mistake about an ulcer of the uterus. Indeed, in the corroding ulcer of the uterus we unfortunately see that this organ is but too capable of taking on all the qualities of ulceration, in the degree only equaled by its extraordinary vitality, the organ being scooped out, or eaten away, in a comparatively short space of time. Cases are also met with, in which the os uteri has been destroyed by the sloughing ulceration and loss of structure, sometimes following the application of the more powerful caustic agents. We are, however, called upon, by the unlimited believers in uterine ulceration, to admit that ulcerative disease may exist for years, in its common form, without any perforation, excoriation, serious loss of substance, or altered configuration. Whether we test the so-called ulceration of the uterus by ulceration occurring in other mucous surfaces, or in the uterus itself, under undoubtedly ulcerative disease, the distinctive characteristics are wanting in the great majority of cases; and they certainly are not found, unless I am most egregiously mistaken, in the enormous proportion of 222 cases of ulceration to 300 cases of promiscuous uterine disease.

In all that I have said, I do not wish it to be supposed that I question the frequency of irritation, chronic inflammation, and sub-acute inflammation, in connection with leucorrhœa. Recent writers would, however, treat leucorrhœa merely and solely as a symptom, not as an independent disorder. But I am well assured that it is often the disease itself, or at least all of it that we can appreciate; and that the irritable or inflammatory condition is excited secondarily, and mainly, by the morbid leucorrhœal secretion. Some change in the innervation or nutrition of the organ occurs; or it sympathizes with a malady in some remote organ, and the secretions are consequently depraved. These depraved secretions irritate the surfaces with which they come in contact, and produce the visible signs of irritation or inflammatory action. We see these discharges sometimes inflame and excoriate even the external integument, but we should never dream of saying that the inflamed condition of the skin was the essential part of the disorder. The same observation applies to the uterus. Thus it is not pathological, nor useful, always to consider leucorrhœa as a mere symptom; and the old plan of astringent injections, though sometimes mischievous, cannot quite be dispensed with, for in some, even profuse leucorrhœas, an astringent injection, by arresting the utero-vaginal discharges, does more than any other plan to soothe inflammatory conditions, or rather, to suspend their causes.

Notwithstanding the use of the speculum,—notwithstanding the use of lamps and glasses, there is often considerable difficulty in ascertaining the precise condition of the cavity of the uterine cervix, engorged as it is, and deep in color from irritation, or other disease, and from the interruption to the circulation in the uterine organs which is almost necessarily dependent on the introduction and expansion of the speculum within the vagina. But in the dead subject no such difficulties exist, and it might certainly be expected, since leucorrhœa is a malady so very common, that uterine ulceration would be frequently revealed by post-mortem examinations. The only place in which, so far as I am aware, post-mortem examinations have been conducted in considerable numbers, with special reference to the determination of the frequency or infrequency of ulceration of the os and cervix uteri, is at St. George's Hospital. For several years past, the condition of the uterus has been examined with great minuteness and accuracy in the dead subject at this hospital.

Mr. Pollock, one of the lecturers on Anatomy at St. George's Hospital, informs me, that for more than three years during which he was Curator to the hospital museum he examined the uterus internally and externally, in all the subjects in the dead-house. During this time, upwards of 100 women died in the hospital annually. In each case, the uterus was laid open, and carefully inspected. Mr. Pollock only detected actual and unmistakable ulceration *in four cases.* Of these, three were scrofulous subjects, and scrofulous ulceration existed in other parts of the body; and in one of them the ulceration involved the vagina extensively as well as the os uteri.

Mr. Gray, who succeeded Mr. Pollock as Curator, informs me that during his curatorship he examined the bodies of 180 women, who died of all diseases in St. George's Hospital, with a distinct view to ascertain the proportion of cases in which ulceration of the uterus existed. These examinations were also conducted with great care and minuteness. Out of the 180 subjects, distinct ulceration of the os and cervix was found in only *three* instances. Slight abrasions, discolorations and granulations were frequently observed, and this accords with the observations of Mr. Pollock. One or two other curators to St. George's Hospital, besides Mr. Pollock and Mr. Gray, have arrived at the same results. It is only by pathological investigations of this kind that we can arrive at infallible results.

But, it may be asked, why bestow so much pains on proving that abrasion, excoriation and ulceration are not *ulceration?* Why dispute as to terms? Simply because a name rules treatment, and because the name of "ulceration" being first given, and heroical treatment not without danger, is frequently resorted to, where milder local applications or constitutional treatment would be equally efficacious. After Mr. Abernethy wrote his celebrated work on the Constitutional Treatment of Local Disease, his idea was pushed to its extreme, and local remedies were often most improperly neglected. Now, in all that relates to the uterine organs, the doctrines of Mr. Abernethy are in danger of being entirely refuted, and we are in some risk of utterly neglecting constitutional treatment and of being entirely absorbed by local applications. This we cannot do without impeding the improvement of the treatment of this class of affections. When a patient is told she has an ulceration of the womb, she often thinks of an ulcer of the leg, or the cheek, &c., and is proportionably frightened, because of the importance of the organ which is the seat of the presumed disease.

There is nothing women will not submit to, to be freed from such a malady. At the present time a veritable uterine panic affects the upper and middle classes of society, and every woman with the slightest ache or discharge, is not satisfied until the peccant organ has been ocularly inspected. I do not believe that this state of things, or its inevitable results, will conduce to the dignity and respectability of our profession. I do not hesitate to affirm, so far as I have eyes to observe and judgment to weigh facts, that much exaggeration prevails respecting the frequency of this same ulceration of the os and cervix uteri—an exaggeration which should be calmed, so that the legitimate methods of examination may lead, not to a suspicion of our profession, but to real improvement in the diagnosis and treatment of uterine disease as it actually exists. We cannot safely repudiate either the local or the constitutional treatment of uterine disease. I have seen cases in which the local ailment has been as far as possible cured; nevertheless, the constitutional symptoms remained unrelieved. I have seen others, in which judicious constitutional treatment has cured the local malady without any topical treatment whatever. But in the combat against disease, we require both constitutional and local weapons; and any views which disparage either the one or the other, must cripple the resources of our art.—*London Lancet.*

